# Migrant health in Italy: a better health status difficult to maintain—country of origin and assimilation effects studied from the Italian risk factor surveillance data

**DOI:** 10.1186/s12963-019-0194-8

**Published:** 2019-11-01

**Authors:** Stefano Campostrini, Giuliano Carrozzi, Santino Severoni, Maria Masocco, Stefania Salmaso, WHO Migration Health Programme, Office of the Regional Director, WHO Regional Office for Europe, Federica Balestra, Santino Severoni, the PASSI National Coordinating group, Sandro Baldissera, Nicoletta Bertozzi, Lara Bolognesi, Pirous Fateh Moghadam, Gianluigi Ferrante, Elisa Quarchioni, Letizia Sampaolo, Maria Masocco

**Affiliations:** 1 Department of Economics, Ca’ Foscari University of Venice, San Giobbe 873, 30121 Venice, Italy; 2 Department of Public Health, Local Health Unit of Modena, Strada Martiniana 21, 41126 Baggiovara, MO Italy; 3 Special Advisor on Health and Migration and Acting Director, Division of Health Systems and Public Health, WHO Regional Office for Europe, UN City Marmorvej 51, DK-2100 Copenhagen, Denmark; 4 National Centre of Epidemiology, Surveillance and Health Promotion, Istituto Superiore di Sanità, Viale Regina Elena 299, 00199 Rome, Italy

**Keywords:** Migrant health, Behavioral risk factor surveillance, Assimilation, Convergence, Country of origin

## Abstract

**Background:**

Many studies on migrant health have focused on aspects of morbidity and mortality, but very few approach the relevant issues of migrants’ health considering behavioral risk factors. Previous studies have often been limited methodologically because of sample size or lack of information on migrant country of origin. Information about risk factors is fundamental to direct any intervention, particularly with regard to non-communicable diseases that are leading causes of death and disease. Thus, the main focus of our analysis is the influence of country of origin and the assimilation process.

**Method:**

Utilizing a surveillance system that has been collecting over 30,000 interviews a year in Italy since 2008, we have studied migrants’ attitudes and behaviors by country of origin and by length of stay. Given 6 years of observation, we have obtained and analyzed 228,201 interviews of which over 9000 were migrants.

**Results:**

While migrants overall present similar conditions to native-born Italians, major differences appear when country of origin or length of stay is considered. Subgroups of migrants present substantially different behaviors, some much better than native-born Italians, some worse. However, integration processes generally produce a convergence towards the behavioral prevalence observed for native-born Italians.

**Conclusions:**

Health programs should consider the diversity of the growing migrant population: data and analyses are needed to support appropriate policies. Many migrants’ subgroups arrive with healthier behaviors than those of their adopted country. However, they are likely to have a less favorable social position in their destination countries that could lead to a change towards less healthy behaviors. Interventions capable of identifying this tendency could produce significant better health for this important part of the future (multicultural) populations.

## Background

Migration has captured the attention of the mass media and the public in response to the massive arrival of migrants, refugees, and asylum seekers into European countries. Some of these migrants are on their way to other non-European destinations; some may eventually return to their home countries, but a large number will probably settle permanently in Europe. Italy has a long history of migration both outbound and inbound. Foreign-born (documented) residents in Italy grew from less than 1% at the turn of the century to almost 10% in the most recent figures [1]. Italy, like many European countries, is clearly facing a transition to a multi-cultural society.

While migration has a number of positive societal effects including economic and employment benefits [2], the recent large-scale population movement has given rise to a number of epidemiological and health system challenges, to which public health and health systems must adjust [3].

Refugees and migrants’ susceptibility to illness is largely similar to that of the rest of the population, although there are substantial differences of health status among different migrant sub-groups, defined, e.g., by countries of origin. Migrants account for a high percentage of the working-age population in low-paid jobs and are more likely to be employed on insecure, temporary contracts [4].

If the recent increasing phenomenon of asylum seekers and refugees has captured the mass media attention for the difficulties that Europe (and other countries) faces in handling this erratic and highly problematic stream of human beings, it should be not forgotten that this is only the tip of the iceberg of a major migration process. In Italy, refugee and asylum seekers were, for HUNCR, 80,000 in 2013 alone (https://www.unhcr.org/551128679#_ga=2.132877774.1587662326.1556791342-1391715842.1556791342), and that number has doubled since, with new and greater challenges in dealing with this “special migrants.” Nonetheless, they are a small percentage (1.6%) among the migrant population that in 2013 was almost 5 million (4,900,000, 8.1% of the Italian population, according to the National Institute for Statistics count). In January 2019, the foreign population in Italy was 5,144,000 (8.5% of the total population), increase due largely to economic migrants. So, refugee migrants have increased exponentially in numbers and needs, but they remain a small percentage of the population, while economic migrants in Italy, as in several other countries, continue to increase at a steady pace (slightly slower after the big recession) and have already started to constitute, numerically, a relevant part of the population.

More information is needed to make health systems more responsive and resilient [5–8] to the needs of these different migration movements. Existing health services have been developed with the needs of the general population in mind, and they may need to be adapted to provide high-quality, accessible, and appropriate health services to migrants and ethnic minorities. These changes must extend to all services (health promotion and education, preventive care and screening, curative and palliative care), and this process requires substantial information.

Among the several questions that could be raised about migrant health, we wanted to address two in particular: the role of country of origin [9–14] and the so-called convergence (assimilation) effect [9, 10, 15–17]. Both are relevant to both design and implement better-targeted public health interventions and, particularly, health promotion actions. Taking this perspective, we limited our study to the foreign-born migrant that settled (or started to settle) in the country, and instead of studying, as in many other published researches, the end-points of mortality and morbidity, we concentrated primarily on risk factors that could be directly influenced both by the “country of origin” and “assimilation” effects.

Another objective of our analyses was to evaluate the use of existing risk factor surveillance systems to study migrant health. Italy has a national risk factor surveillance system, PASSI (Progresses in Health in the Italian Local Health Units) [18]. Recently, these data have been used to study migrants’ subgroups [19], focusing—because of surveillance system eligibility criteria—on documented migrant population resident in Italy. The analysis of these data, unique for sample size and approach, can offer an in-depth view of migrants’ health, attitudes, and behaviors, although limited to those migrants relatively more integrated. Here, we present some further analysis using information on migrant respondent length of stay in Italy to examine how risk factors changed over time with the integration process that, as observed, is not always favorable towards a better health outcome. Results also show how prevention of chronic diseases in the general population should be tailored to specific ethnic behaviors, given the evidence of strong variability between migrant groups defined by country of origin. The paradigmatic analyses here discussed can show how risk factor surveillance systems are potentially capable of informing advanced policies on migrants’ health, often advocated [20] and, to date, rarely practiced.

## Methods

Data have been collected between 2008 and 2013 through the ongoing surveillance system for prevention of non-communicable diseases (NCDs) called PASSI. Two hundred twenty-eight thousand two hundred one interviews have been analyzed.

The system collects over 3000 interviews per month (every month, an independent sample is drawn) from approximately 90% of the Italian Local Health Units (LHUs), the administrative organization of the National Health System responsible for most of the health services offered to the population. During the period of observation, the surveillance system had coverage of over 90% of the Italian population and a response rate of over 85%. Given the peculiarities of a system that continuously collects data, non-responses have been taken into account through field substitution [18, 21]. Further methodological information can be found in Baldissera and colleagues [22] and other published papers [23–25].

Interviewees are randomly sampled from the list of the resident population aged 18–69, registered at each LHU. Subjects capable of sustaining a conversation in Italian were considered eligible for the interviews. In Italy, the registration at the LHU is necessary to access all the services that are universally offered by the national health system. Consequently, among the settled migrants, those included in the study are those that have at least started an integration process (and will be likely reachable by public health and health promotion programs).

In our study, the respondents with foreign citizenship were considered as migrants. As for the country of origin, we have aggregated these into a few big geographical areas for representative purposes and accepting limitation of merging countries with different cultures. Area of origin was classified as follows: European Union (EU), other European countries, North Africa, Sub-Saharan Africa, Asia, and America. The 600 respondents included in the initial sample, coming from high-income countries (as defined by the World Bank—mainly from EU and North America), have not been considered in the present analyses.

The Italian surveillance system covers several main health-related topics, organized in four major areas: wellbeing and mental health, risk factors, adherence to preventive programs, and safety. For this presentation, we have selected a few variables and statistics, choosing those on which precedent analyses showed more differences among the migrants’ population, covering a mental health and a wellbeing indicator, the major behavioral risk factors, and a measure of prevalence of access to preventive services. The aim is to show differences among migrants and Italians, and within the migrant groups to study the country of origin effect. We also propose further analyses to test the effect of assimilation process, taking as indicator the length of stay in Italy.

The variables analyzed are:

- *Perception of health status*: answers given to the question “How is your health in general?” with five answer categories: very good, good, fair, bad, very bad (Table 1 shows the percentage of those responding in the first two categories);
- *Symptoms of depression*: the prevalence of population with symptoms of depression is evaluated on the basis of a two-question validated test known as “Patient Health Questionnaire 2 (PHQ-2)” [26]; general results on the PASSI application have been presented elsewhere [24, 27].
- *Prevalence of smokers*: those that report to have smoked at least 100 cigarettes in their own life and that are current smokers;
- *Prevalence of higher risk alcohol drinkers*: if respondents were at least in one of the following categories: heavy drinkers (15 or more drinks a week for men, and 8 for women), and/or alcohol drinkers mainly outside of meals, and/or binge drinkers (5 drinks or more in one occasion for men, and 4 for women);
- *Prevalence of overweight and obese*: defined on self-reported height and weight and the derived body mass index (BMI) with the usual thresholds (between 25 and 29.9 for overweight and 30 and over for obese);
- *Prevalence of (correct) participation in preventive cervical cancer screening*: percentage of women in the appropriate age groups that reported a Pap smear test/HPV test at least once in the last 3 years.

**Table 1:** Distribution of foreign population resident in Italy (age 18–69) interviewed in the surveillance system PASSI (2008–2013; *n* = 228,201; foreigners 9516, 4.1% of the respondents) by selected sociodemographic variables. Comparison with residents with Italian citizenship

	% on the total of migrants	CI	% among Italians	CI
Country of origin (geographical area)*				
European Union (not From HDC)	31.2	30.1–32.2%	–	–
Other European countries	28.9	27.9–29.9%	–	–
North Africa	12.7	11.9–13.5%	–	–
Sub-Saharan Africa	6.3	5.7–6.9%	–	–
Asia	10.4	9.7–11.2%	–	–
America (not from HDC)	10.6	9.8–11.4%	–	–
Length of stay in Italy				
0–4 years	15.7	14.7–16.7%	–	–
5–9 years	29.7	28.5–31.0%	–	–
10 years and over	54.6	53.3–55.9%	–	–
Gender				
Male	41.4	40.2–42.6%	49.8	49.7–49.9%
Female	58.6	57.4–59.8%	50.2	50.1–50.3%
Age				
18–24	11.3	10.6–12.0%	11.1	10.9–11.3%
25–34	31.7	30.5–32.8%	17.9	17.7–18.0%
35–49	42.2	41.1–43.4%	34.5	34.4–34.6%
50–69	14.8	14.0–15.7%	36.5	36.4–36.6%
Economic difficulties				
A lot	25.4	24.4–26.5%	14.2	14.0–14.4%
Some	48.2	47.0–49.4%	41.4	41.1–41.7%
None	26.4	25.3–27.5%	44.4	44.1–44.7%
Education				
Elementary-no school	9.7	9.0–10.5%	10.2	10.0–10.3%
Middle school diploma	34.0	32.9–35.2%	30.1	29.8–30.3%
High school diploma	45.3	44.1–46.5%	45.0	44.8–45.3%
University degree	11.0	10.2–11.8%	14.7	14.5–14.9%
Marital status				
Single	26.0	24.9–27.1%	32.7	32.5–32.9%
Married	64.8	63.6–65.9%	60.0	59.6–60.1%
Separated/divorced	6.6	6.1–7.3%	4.9	4.8–5.0%
Widow/er	2.6	2.3–3.1%	2.5	2.4–2.6%

*Only citizens from countries with high migration pressure have been considered, those coming from highly developed countries (HDC), 600 in our sample, less than 0.3%, have been excluded by the analysis

For descriptive statistics and sub-group comparison, confidence intervals are computed at the 95% level. When comparison is made on standardized data/prevalence, a direct standardization by gender and age has been performed using as reference the Italian population resulting from the National Official Statistics.

A logistic regression analysis has been performed using STATA software to compute, for each relevant variable, odds ratios (ORs) of migrants and migrant sub-groups (defined by the country of origin) in comparison with native Italians as a reference group. Possible interactions with other factors have been included in the model and are presented as *conditional* ORs. For each variable of interest, the ORs of comparison groups are computed, given possible effects of other variables in the model. These are sex, age, and the presence of economic difficulties, as a proxy indicator of social-economic class. The latter categorizes respondents based on the answer to the question “Considering all the resources of your family, how do you get to the end of the month? – very easily; rather easily; with some difficulties; with a lot of difficulties”.

## Results

Table 1 shows the basic demographics of migrant residents in Italy. Statistics from the surveillance system PASSI are similar to those provided by ISTAT (National Institute of Statistics). Migrants are younger than the rest of the Italian population and have a higher presence of women than men. This can be explained by the recent phenomenon of the caregivers (“*badanti*”), which are mostly women, providing assistance to elderly or disabled persons at their home. Their presence has greatly increased in the last decades and is attributed to the aging of the population, Italy has the second oldest longevity in the world, and to the insufficient number and high costs of sheltered houses. These caregivers often come from the Eastern European countries, both from the European Union and outside EU, and this partially explains why the most frequent areas of origin of migrants are European. Among migrants, there are more married people. This may support a rise in the Italian fertility rate. Italy had one of the lowest fertility rate in the world, 1.3 in the year 2000, while in the 1970s, it was higher than 2. It increased only when the immigration process started at the turning of the century: the most recent figures are higher than 1.4 [28].

The migrants’ socio-economic status is generally lower than those of native-born Italians. While they present a similar educational level, but with a slightly lower percentage of those with university degree, important differences can be found in the self-reported economic status. Migrant groups have reported more economic difficulties than Italians, with some differences by country of origin (data not shown), similarly with education.

Table 2 presents a direct comparison of prevalence between Italian and foreign residents, standardized by age and gender (in brackets), and not. These two measures are both informative: the latter compares the actual differences among residents, offering numerical values potentially useful for policy development. The standardized comparison offers insight into the difference in attitudes and behaviors between population sub-groups, independently by possible differences caused by gender and/or age structure.

**Table 2:** Comparison between Italian and foreign born population resident in Italy (age 18–69) interviewed in the surveillance system PASSI (2008–2013; *n* = 228,201) by selected health-related variables and country of origin (showing only those with the better and worse situation)

	Italian*	CI	Migrants*	CI	Better	%	CI	Conditional OR**	CI	Worse	%	CI	Conditional OR**	IC
Perception of health status (good + very good)	67.7% (68.0%)	67.4–67.9% (67.8–68.2%)	76.4% (73.2%)	75.3–77.4% (71.9–74.6%)	Asians	80.3% (77.8%)	76.6–83.7% (73.8–81.9%)	1.62	1.27–2.07	Italians	67.7% (68.0%)	67.4–67.9% (67.8–68.2%)	–	–
Symptom of depression	6.7% (6.7%)	6.6–6.8% (6.5–6.8%)	6.0% (6.1%)	5.5–6.6% (5.3–6.8%)	Asians	3.1% (3.1%)	1.9–5.0% (1.4–4.8%)	0.44	0.27–0.72	North Africans	8.2% (10.1%)	6.4–10.4% (6.5–13.6%)	1.18	0.90–1.55
Prevalence of smokers	28.4% (28.5%)	28.2–28.6% (28.3–28.8%)	28.8% (29.1%)	27.7–29.9% (27.8–30.4%)	Sub-Saharan Africans	12.1% (12.7%)	9.2–15.7% (8.6–16.8%)	0.25	0.18–0.34	Foreigners from EU	39.9% (41.7%)	37.8–42.0% (38.8–44.5%)	1.6	1.46–1.76
Prevalence of higher risk alcohol drinker	16.8% (16.8%)	16.6–17.0% (16.6–17.0%)	14.5% (14.4%)	13.7–15.4% (13.4–15.3%)	North Africans	6.6% (5.7%)	5.1–8.6% (4.0–7.5%)	0.3	0.23–0.39	Americans	21.9% (22.7%)	18.8–25.4% (19.1–26.4%)	1.62	1.34–1.96
Prevalence of overweight and obese	42.1% (41.7%)	41.8–42.3% (41.5–42.0%)	39.2% (44.1%)	38.0–40.4% (42.6–45.6%)	Asians	31.4% (30.2%)	27.5–35.5% (25.9–34.6%)	0.66	0.53–0.83	North Africans	44.9% (50.1%)	41.6–48.3% (46.0–54.3%)	1.1	0.94–1.29
Prevalence of a correct attendance to preventive cervical cancer screening	76.7% (76.5%)	76.4–77.1% (76.1–76.9%)	70.0% (69.3%)	68.3–71.6% (67.3–71.2%)	Americans	80.7% (79.4%)	76.5–84.4% (74.9–83.9%)	1.31	1.02–1.70	Asians	52.2% (51.7%)	45.5–58.8% (43.9–59.5%)	0.36	0.27–0.48

*In brackets are prevalence standardized by gender and age

**Conditional ORs are computed through a logistic regression including the following variables: gender, age, economic status (difficulties to get at the end of the month). Italian have been taken for reference in ORs computation

To appreciate the differences among countries of origin, and for the sake of brevity, for each variable considered, the geographical group presenting the higher and the lower prevalence is reported in the table. Conditional OR measures offer an estimate of the net effect of being migrant, from that specific area, controlling for some possible confounders.

As it is quite evident from the table, the migrant population as a whole appears not too different in terms of health attitudes and behaviors from Italians. The only significant differences are a better perception of health status among migrants and a lower attendance of preventive services. Results for the cervical cancer screening, in Italy offered free of charge to all the women over 18, are similar (data not shown) also for the other two major preventive screenings offered in Italy universally: mammography and colorectal screening.

If migrants overall present figures very similar to the Italians, the differences inside of the migrant population are relevant, confirming that the country of origin has a major influence on health behaviors and attitudes. Notably, Italians never appear as the better subgroup, and even more interestingly, when possible socio-demographic differences have been taken into consideration, the better migrant subgroup present ORs significantly better than Italians. Another interesting result comes from the sub-group health profile: the abovementioned differences do not derive from a single sub-group of population that is always better (or worse) with regard to the health variables here considered. Asians, for instance, have better results on several variables, but they are much worse in the attendance of preventive services. In this, Americans are instead even better than Italians for preventive services, but the worst population subgroup for risky alcohol behaviors.

Table 3 presents the same prevalence broken down by gender. As it can be seen, gender seems to play a similar role among Italians and migrants: where the prevalence is higher for women than men among Italians and is higher for women among migrants and vice versa. Interesting, the case of smoking, where the gender differences are emphasized among migrants, reports male migrants a much higher prevalence of smokers than Italian males and female migrants a lower prevalence compared to Italian females.

**Table 3:** Comparison between Italian and foreign born population resident in Italy (age 18–69) interviewed in the surveillance system PASSI (2008–2013; *n* = 228,201) by gender

	Italian males*	CI	Italian females*	CI	Migrants males*	CI	Migrants Females*	CI
Perception of health status (good + very good)	71.9% (72.2%)	71.5–72.2% (71.9–72.6%)	63.4% (63.8%)	62.9–63.7% (63.5–64.2%)	80.3% (77.2%)	78.8–81.9% (75.0–79.3%)	73.7% (69.3%)	72.2–75.1% (67.7–71.1%)
Symptom of depression	4.6% (4.5%)	4.4–4.7% (4.4–4.7%)	8.9% (8.8%)	8.7–9.1% (8.6–9.1%)	4.7% (4.7%)	3.9–5.6% (3.7–5.8%)	6.9% (7.2%)	6.1–7.6% (6.2–8.2%)
Prevalence of smokers	32.9% (33.1%)	32.5–33.2% (32.7–33.5%)	23.9% (24.0%)	23.6–24.3% (23.7–24.4%)	39.0% (38.0%)	37.1–40.8% (35.7–40.4%)	21.6% (20.5%)	20.3–22.9% (19.1–21.9%)
Prevalence of higher risk alcohol drinker	22.6% (22.7%)	22.3–22.9% (22.4–23.0%)	11.2% (11.3%)	11.0–11.5% (11.1–11.5%)	22.0% (19.7%)	20.4–23.6% (18.0–21.5%)	9.2% (8.9%)	8.3–10.1% (7.9–9.9%)
Prevalence of overweight and obese	51.2% (51.0%)	50.8–51.6% (50.6–51.4%)	32.8% (32.5%)	32.5–33.2% (32.1–32.8%)	46.7% (49.5%)	44.8–48.6% (47.1–51.9%)	33.8% (38.8%)	32.3–35.3% (37.0–40.6%)

*In brackets are prevalence standardized by age

Table 4 presents the effect of the length of stay (less than 5 years, from 5 to 9, 10 or over) as a proxy for the integration process and as a potential modifier of migrants’ attitude and behaviors. We acknowledge that other factors not measured in the PASSI surveillance system could affect the integration process. The only additional available variable that could play an important role in the progress of integration is the country of origin, which we have seen being so relevant for the variables examined. Unfortunately, the relatively small sample size of each group of foreigners did not allow stratification by length of stay and country of origin. In the future, a larger database of the PASSI system providing a larger sample for migrants will allow also these analyses.

**Table 4:** Comparison between Italian and foreign born population resident in Italy (age 18–69) interviewed in the surveillance system PASSI (2008–2013; *n* = 228,201) by selected health-related variables and length of stay

	Italians*	CI	Migrants from 10 years or over*	CI	Migrants from 5 to 9 years*	CI	Migrants from less than 5 years*	CI
Perception of health status (good + very good)	67.7% (68.0%)	67.4–67.9% (67.8–68.2%)	74.3% (73.4%)	72.6–76.0% (71.5–75.2%)	77.9% (75.0%)	75.8–79.9% (71.5–78.5%)	83.3% (75.8%)	80.7–85.6% (71.0–80.5%)
Symptom of depression	6.7% (6.7%)	6.6–6.8% (6.5–6.8%)	6.7% (6.5%)	5.8–7.8% (5.4–7.5%)	5.9% (5.2%)	4.8–7.1% (3.8–6.6%)	4.3% (4.1%)	3.1–5.9% (2.3–5.9%)
Prevalence of smokers	28.4% (28.5%)	28.2–28.6% (28.3–28.8%)	28.3% (28.6%)	26.7–30.0% (26.8–30.4%)	30.2% (33.2%)	28.0–32.5% (29.7–36.7%)	25.1% (25.8%)	22.2–28.1% (21.5–30.0%)
Prevalence of higher risk alcohol drinker	16.8% (16.8%)	16.6–17.0% (16.6–17.0%)	15.9% (16.3%)	14.6–17.4% (14.9 –17.8%)	15.0% (16.5%)	13.4–16.9% (13.6–19.5%)	14.5% (14.6%)	12.3–17.1% (11.5–17.6%)
Prevalence of overweight and obese	42% (41.7%)	41.8–42.3% (41.5–42.0%)	43.1% (44.4%)	41.2–45.0% (42.4 –46.4%)	35.6% (41.9%)	33.2–38.0% (38.2–45.6%)	30.6% (41.0%)	27.5–33.8% (35.6–46.3%)
Prevalence of a correct attendance to preventive cervical cancer screening	76.7% (76.5%)	76.4–77.1% (76.1–76.9%)	78.1% (77.2%)	75.7–80.3% (74.7–79.8%)	73.1% (70.2%)	70.1–75.9% (66.5–74.0%)	57.9% (61.2%)	53.1–62.6% (54.3–68.1%)

*In brackets are prevalence standardized by gender and age

Despite these limitations, overall results appear quite interesting. The migrant respondents seem to be more similar to the Italians as the length of stay increases, confirming what has been called the “convergence effect” or the “assimilation process” [9, 10, 15–17, 29]. Moreover, the results are certainly interesting for health policies, such as recognizing the increased participation in preventive actions and assessing negative increases in risk factors, as already described by Turrin et al. [30].

## Discussion

The present study is unique, with a large population-based sample size and a risk factor approach. As with every study, there are limitations, but these do not ultimately impair the conclusions.

The main limitation of our study has been to consider only migrants who were relatively more integrated: those registered in the National Health System and able to sustain a conversation in Italian. Furthermore, cultural differences and linguistic problems could have affected migrants’ answers; this is particularly relevant in a study that relies on self-reported responses. To better understand the range of the abovementioned limitations, the migrants covered by the PASSI surveillance are between 75 and 85% of those present in Italy. Estimates are difficult, since the presence of undocumented migrants is almost impossible to identify. Recent research estimates argue that around 5 to 10% of migrants to Italy may be undocumented [31, 32]. Some additional 20% may be lost because they are unreachable or not capable of a conversation in Italian. A recent survey estimated that one third of the foreign population present in Italy have problems with the Italian language—www.istat.it/it/files//2014/07/diversità-linguistiche-imp.pdf. All this considered, we can reasonably assert that our study is relevant, from a numerical point of view, as it likely includes more than two thirds of migrants present in Italy at the time of the study. It is worthwhile to note that those not included in the study are hard to reach groups also for preventive programs and are likely to have limited access to health services. Therefore, one needs to keep in mind the guidance role of surveillance [33] and realize that many public health and health promotion interventions, particularly in the NCDs area with long-term effects, can be targeted only to documented migrants. Nonetheless and in contrast to most of the published studies which analyzed morbidity and mortality and late endpoints from prevention actions, our study focuses on measures relevant for setting preventive public health policies and reducing inequalities among population sub-groups.

If it is acknowledged that all the migrants are not the same, the data and analyses presented here show quite clearly that among the more settled groups, the differences in health behaviors and attitudes are substantial. Earlier research has shown the importance of observing the country of origin as a key variable [9–14], but most of these studies were focused on a few migrants’ groups and looked specifically at health outcomes (morbidity and mortality). Our data show the relevance of differences in health attitudes and behaviors by country of origin. These differences appear substantial, particularly when possible socio-demographic differences have been taken into account.

The highly composite puzzle may be explained by cultural differences and raises a public health problem: migrant populations cannot be considered as a homogenous entity. There could be some migration effects shared by all, but, from our data, it looks like the country of origin has a much relevant power in influencing health attitudes and personal behaviors. Country of origin is certainly only a proxy of many factors influencing health and behaviors, such as cultural and religious values, dietary habits, and health concepts, but eventually, these latter types of variables are the ones to be considered for public health policies.

Combining the results from the analysis by the area of origin with those from the length of stay allows the findings to be potentially useful for policy making and health promotion interventions. In our opinion, the culture of the country of origin is more important than to the notion of healthier migrant selection [14, 34]. That is, the present migrant population, after a few years in Italy, has generally better health behaviors and attitudes than the general Italian population. Migrants perceive better health, are less depressed, smoke less, drink less, and report a lower prevalence of obesity/overweight. However, length of stay relates to worsening figures. Earlier research has shown how convergence of health levels toward the local population is quite typical for migrants [9, 10, 17, 35, 36], although not always [12], or it can be a slow process [36]. What we found in the variables and in the population considered is that a convergence process is certainly taking place. In our opinion, this is not just the effect of a “natural” assimilation. Quite often, migrants share the conditions attributed to the lower socio-economic classes [37], and in Italy, as in many countries, most of the health-related variables are highly influenced by socio-economic levels. To quote a figure from the PASSI surveillance, prevalence of smoking in the adult population, for instance, is almost twice as much in the lower economic class than in the upper one (respectively around 40% and 20%). Consequently, the assimilation process could be harmful for migrants, and this could explain the worse health levels in the elderly population found among migrants by Solé-Aurò and Crimmins in a previous European study [38].

Our results are not necessarily bad news for public health. Public health has some powerful tools to overcome these difficulties, and the data on cervical cancer screening provide a nice example of the effects of health promotion applied to a preventive service. Pap smear tests have been free of charge and universally offered in all Italy for several decades, but the overall coverage increased, and socio-economic differences started to decrease [39], when a health promotion approach has been taken involving all the population in the screening activity, better targeting the promotion of these services [30]. Similar results, interestingly, are seen also among the migrant population: the prevalence of migrant women attending cervical screening increases as integration (and exposure to health promotion) increases. This can be due to better targeted interventions, which used linguistic and cultural “translations,” thanks also to the involvement of cultural mediators working with the preventive department staff.

## Conclusions

A tentative conclusion is that, when a health promotion approach is taken, the generally negative combination of migration and lower socio-economic status can be effectively reduced or eliminated (cf. Table 4).

Combining this observation with the results coming from the analyses by the country of origin allows one to conclude that migrants have great potential for increasing the overall population health of the country of immigration, at least with regard to NCDs: they arrive generally with better health attitudes and behaviors, some (depending from the country of origin) substantially better. Results presented here argue for appropriate policies on migrant health: for example, those that would take into consideration cultural differences as an opportunity and not as a limitation when actions are taken.

The great migration movement asks for non-emergency measures capable of adjusting health systems to answer the needs of both arriving or transiting migrants and settled new citizens. Current migration figures underscore the fact that the influx of refugees, asylum seekers, and migrants is not an isolated crisis but an ongoing challenge [4] that will affect European and many other countries for some time to come, with medium- and longer-term security, economic, and health implications[2]. To make health systems more resilient to this major change in the populations’ structure, information is needed [7, 9, 40]. This study illustrates the valuable approach arising from the wise use of extant behavioral risk factor surveillance systems data.

## Data Availability

PASSI surveillance data can be accessed at http://www.epicentro.iss.it/passi/ The dataset used for the analyses is not publicly available, for specific policy of the National Institute of Health and of the Italian Ministry of Health, but are available by the National Institute of Health on reasonable request.

## Abbreviations

BMI: Body mass index
EU: European Union
LHU: Local Health Unit
NCDs: Non-communicable diseases
ORs: Odds ratios
PASSI: Progresses in Health in the Italian Local Health Units –LHU
